# Single-cell transcriptome analysis reveals status changes of immune cells in chronic kidney disease

**DOI:** 10.3389/fmed.2024.1434535

**Published:** 2024-12-03

**Authors:** Xinhuan Fan, Yuxin Zhu, Hao Kan, Aiqin Mao, Li Geng, Changzhu Li, Ka Zhang

**Affiliations:** ^1^Department of Urology, Lu'an Hospital of Anhui Medical University, Lu'an, China; ^2^Wuxi School of Medicine, Jiangnan University, Wuxi, China; ^3^School of Food Science and Technology, Jiangnan University, Wuxi, China

**Keywords:** human kidney, CKD, scRNA-seq, immune cell, SPP1 macrophages

## Abstract

**Background and aims:**

The immune system plays a crucial role in the development of kidney diseases. Chronic kidney disease (CKD) can lead to various complications, potentially affecting multiple systems throughout the body. Currently, the description of the immune system in human CKD is not comprehensive enough. Constructing a CKD kidney atlas using single-cell RNA sequencing (scRNA-seq) can provide deeper insights into the composition and functional changes of immune cells in CKD, facilitating the discovery of new therapeutic targets.

**Methods:**

We processed and integrated scRNA-seq datasets from healthy and CKD kidneys from three independent cohorts using the same approach (including 42 normal samples and 23 chronic kidney disease samples). Subsequently, we conducted gene enrichment and intercellular communication analysis to construct an immune cell atlas of the kidneys in CKD patients.

**Results:**

We identified nine major kidney cell clusters. Further clustering analysis of different immune cell clusters revealed that, compared to normal kidneys, CKD patients’ kidneys had decreased CD16+ NK cells while CD4+ naive helper T cells and CCR7+ DC increased. Partial activation of the WNT signaling pathway was observed in T cells and NK cells of CKD patients, while some metabolism-related genes were inhibited. Myeloid cell subgroups also exhibited abnormal signaling pathway alterations. Additionally, we discovered a unique population of SPP1 macrophages in CKD, which are recruited by chemokines released from aPT and aTAL cell subpopulations. These SPP1 macrophages may promote cellular fibrosis through the signaling of SPP1, FN1, and various receptors.

**Conclusion:**

We established a human CKD kidney immune cell atlas and identified SPP1 macrophages as a unique cell type in CKD. The interaction between SPP1 macrophages and damaged cells may serve as a potential therapeutic target for treating CKD in the future.

## 1 Introduction

Chronic kidney disease (CKD) is a condition characterized by chronic structural and functional impairments of the kidneys due to various reasons, leading to a gradual loss of kidney function over time. Currently, CKD is a global public health concern, affecting approximately 10% of the world’s population, with its incidence on the rise worldwide (1). The Global Burden of Disease Consortium predicts that by 2040, CKD will be one of the top five diseases contributing to reduced life expectancy (2). However, understanding of CKD remains inadequate, and treatment options are limited at present.

As CKD progresses, both the function and structure of the kidney undergo corresponding changes, ultimately leading to end-stage glomerular closure and tubulointerstitial fibrosis. The role of immune cells in CKD is of paramount importance. One of the most significant changes observed is the influx and expansion of immune cells (3). In CKD, damaged cells release a variety of cytokines to attract immune cells, which, once recruited, release pro-inflammatory cytokines (3, 4). These immune cells become excessively activated within the kidney, thereby further accelerating the progression of fibrosis (5). The advent of single-cell RNA sequencing (scRNA-seq) and spatial transcriptomics has yielded unparalleled insights into the molecular and cellular composition of healthy mouse and human kidneys, including changes during development and disease (6–11). However, current scRNA-seq studies on the cellular composition and functional changes in CKD have primarily focused on proximal tubules and epithelial cells (10, 11). A systematic characterization of the composition and functional changes of immune cells in CKD remains lacking.

Therefore, we integrated and analyzed three independent kidney scRNA-seq datasets to create a detailed immune cell atlas of the kidneys in CKD patients. By comparing differential gene expression ratios, gene enrichment, and cell communication analysis, we identified changes in the composition and function of renal immune cells under physiological and pathological conditions. This exploration aimed to elucidate the impact of immune cells on CKD development, identify signaling pathways associated with these changes, and provide potential therapeutic targets for CKD treatment.

## 2 Methods

### 2.1 scRNA-seq data acquisition

The normal and CKD scRNA-seq datasets were obtained from published studies based on human kidney tissue. This study utilized the following human kidney scRNA-seq datasets as controls or CKD samples: Kuppe et al. (normal group = 9, CKD group = 6), (11) Lake et al. (normal group = 20, CKD group = 17) (10), Stewart et al. (normal group = 13) (12). The datasets were downloaded from the respective repositories using the provided accession numbers.

### 2.2 scRNA-seq data processing and clustering

scRNA-seq expression data analysis was performed using the R package Seurat (version 4.0.6) (13). A Seurat object list was established for each sample across diverse datasets. The DoubletFinder tool was utilized to remove potential doublets (14). Quality control measures were established by determining cutoff values derived from the distributions of each variable across the entire dataset. Cells were filtered for nFeature_RNA > 200 and nCount_RNA < 12,000 and mitochondrial reads < 30%. We removed ribosomal genes due to their strong influence on downstream clustering (15). Subsequently, each dataset was integrated using the scVI algorithm to remove batch effects between different samples (16). Cells were clustered based on a graph-based clustering approach in the FindNeighbors (top 30 PCA dimensions) and FindClusters functions (ranging from 0.2 to 1.2), then the clustree (17) (v0.4.3) R package was used to select an appropriate resolution by assessing cluster stability. Finally, we obtained 32 clusters with resolution = 0.5. Marker genes for individual clusters were determined computed by FindAllMarkers function and the UMI count was a latent variable. Cell types were annotated using CellTypist and custom gene lists, “Adult_Kidney_HCAKidney2022.pkl” was used as the reference dataset (18). The second round of clustering procedures for immune cells was the same as above.

### 2.3 Differential proportion analysis

We performed differential proportion analysis to assess shifts in cell subpopulation proportions between normal and CKD conditions, following the approach of Farbehi et al. (19). Cells were clustered across both conditions and assigned group (G) and cluster (L) labels. For each cluster, a statistic was computed, representing the difference in proportions between the conditions. To assess significance, a null distribution was generated by permuting cluster labels for 10% of cells (*w* = 0.1) across 100,000 iterations. Empirical *p*-values were calculated, and a Bonferroni correction was applied to control for multiple testing, with significant changes defined at *p*-value < 0.05.

### 2.4 Single-cell gene enrichment analysis

Single-cell gene set enrichment analysis was conducted using the VISION (v2.1.0) R package, following the instructions provided in the package’s documentation. Hallmark and KEGG gene sets were obtained from the Molecular Signatures Database (MSigDB) and used to calculate enrichment scores for each cell in the dataset (20, 21). These enrichment scores, reflecting pathway activity, were incorporated into Seurat objects for downstream analysis. For visualization, the enrichment scores were mapped onto the cells using the FeaturePlot function of Seurat, with max.cutoff and min.cutoff set to highlight the most relevant score ranges. To compare enrichment levels across different cell types or subtypes, the AverageExpression function was employed, averaging enrichment scores within each group. Heatmaps displaying these average scores were generated using the pheatmap package, providing a clear visualization of pathway activation patterns across various cell populations.

### 2.5 Cell communication analysis

Cell–cell signaling pathways between each identified cell cluster were comprehensively analyzed using CellChat (version 1.5.0), a tool specifically designed for the systematic analysis of cell–cell communication based on ligand-receptor interactions (22). Prior to the analysis, immune and non-immune cell Seurat objects were merged into a single integrated dataset to enable holistic investigation of intercellular communications across all cell types. For the inference of cell–cell communication networks, we focused exclusively on the secreted signaling interaction category from CellChat’s manually curated ligand-receptor interaction database, which encompasses experimentally validated protein–protein interactions and pathway annotations. The analysis pipeline included several key steps: First, expression data was preprocessed and normalized according to CellChat’s requirements. Subsequently, potential ligand-receptor interactions were identified based on the expression levels of signaling molecules. Statistical significance of these interactions was assessed using a probability threshold of 0.05. The communication pattern analysis was performed using CellChat’s built-in statistical approaches, including differential expression analysis and permutation tests. All other parameters in the CellChat algorithms were maintained at their default settings to ensure reproducibility.

We employed NicheNet to identify ligand-receptor interactions between injured cells and enriched immune cells. Initially, we identified the intersection of differentially expressed genes between aPT, aTAL, and normal cells, selecting genes with log2FC > 0.5 and adjusted *p*-value < 0.05 as the genes of interest. All expressed genes in the enriched immune cells were used as the gene background, where a gene was considered expressed if it had non-zero values in at least 10% of cells within a cell type. Furthermore, aPT and aTAL were designated as sender cells, while the enriched immune cells were designated as receiver cells. Ligands expressed by sender cells were ranked based on Pearson correlation coefficients between ligand target predictions and observed transcriptional responses. Receptor cells were inferred based on NicheNet’s pre-built prior model, which leverages multiple curated ligand-receptor and signaling databases to infer interactions between sending ligands, receiving receptors, and downstream target genes.

### 2.6 Deconvolution analysis

To further investigate the relationship between SPP1+ macrophages and injured kidney cells, we employed the cell2location method, which was reported as the top-performing tool for this task in a recent benchmarking study. The Visium Spatial Gene Expression slide from Lake et al. (10). Briefly, cell2location is a Bayesian model that estimates the abundance of each cell population at each location by decomposing mRNA counts in Visium data using transcriptional signatures of reference cell types. Initially, we applied the negative binomial regression model implemented in cell2location and estimated the reference signatures of our annotated cell types based on scRNA-seq data. The regression model for single-cell data was initialized using batch as the batch_key, and the model was trained for a maximum of 250 epochs. Subsequently, for spatial transcriptomics data, we retained genes shared with scRNA-seq and initialized the regression model using single-cell reference signatures, default settings, and hyperparameters recommended by cell2location. Through manual visual inspection, we estimated eight cells per spot and accordingly set N_cells_per_location to 15. The model was then trained for a maximum of 30,000 epochs. We plotted the ELBO loss history during training and evaluated mapping quality by examining reconstruction accuracy plots. Additionally, we utilized Scanpy’s plotting function scanpy.pl.spatial to visualize spatial scatter plots of cell type abundance in spatial coordinates.

## 3 Results

### 3.1 Single-cell profiling of chronic kidney disease

**Figure 1:**
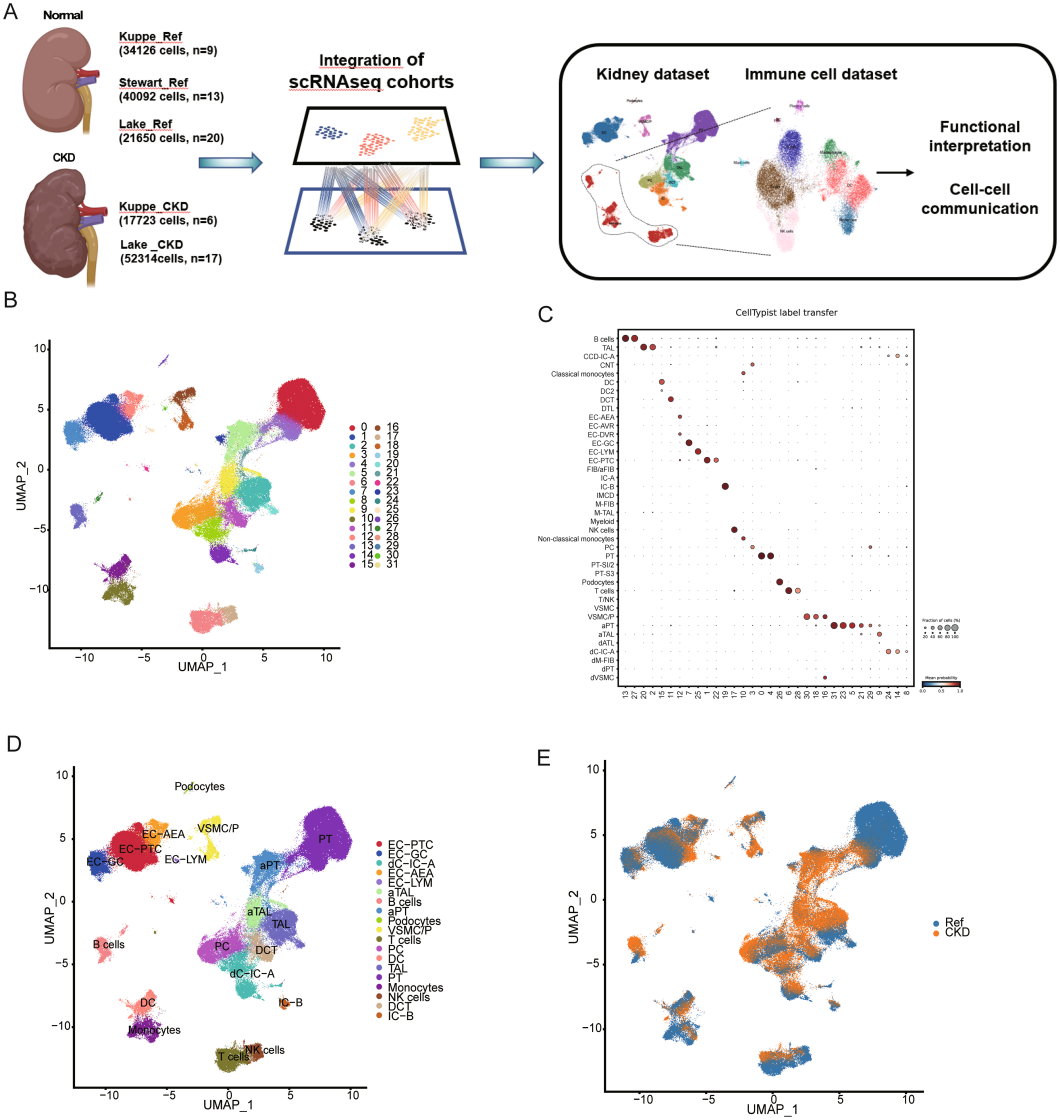
Integrated single-cell atlas of human normal and CKD kidney. **(A)** Graphic overview of this study design. The sample comprises scRNA sequencing data from 42 normal kidneys and 23 CKD kidneys obtained from three independent studies. **(B)** Uniform manifold approximation and projection (UMAP) visualization of 165,905 normal and CKD kidney cell atlases. **(C)** The prediction of cell type annotations using CellTypist. **(D)** UMAP plots for different cell types. The colors of the cells represent different cell types. **(E)** UMAP plots of cell clusters from different conditions.

For a systematic evaluation of immune cell changes in chronic kidney disease, we integrated three independent scRNA-seq datasets using scVI, comprising a total of 65 samples, including 42 normal samples and 23 CKD samples (Figure 1A). The collected scRNA-seq data underwent the same processing pipeline for reprocessing. After strict quality control filtration, we retained 165,905 cells (95,868 normal; 70,037 CKD) for subsequent analysis. Using unsupervised clustering and after visualization by uniform manifold approximation and projection (UMAP), we identified 32 distinct cell clusters (Figure 1B). We annotated cell types using CellTypist and classical marker genes (Figure 1C; Supplementary Figure S1B), resulting in 9 major cell types and 19 subclasses, including proximal tubule (PT), endothelial cells (EC), immune cells, podocytes (Pod), ascending thin limbs (ATL) and distal convoluted tubule cells (DCT) (Figure 1D; Supplementary Figure S1A). Among them, adaptive proximal tubule (aPT) and adaptive ascending thin limb cells (aATL) are predominantly expressed in CKD samples (Figure 1E).

### 3.2 Changes in kidney T cell and NK cell composition and function in CKD

**Figure 2:**
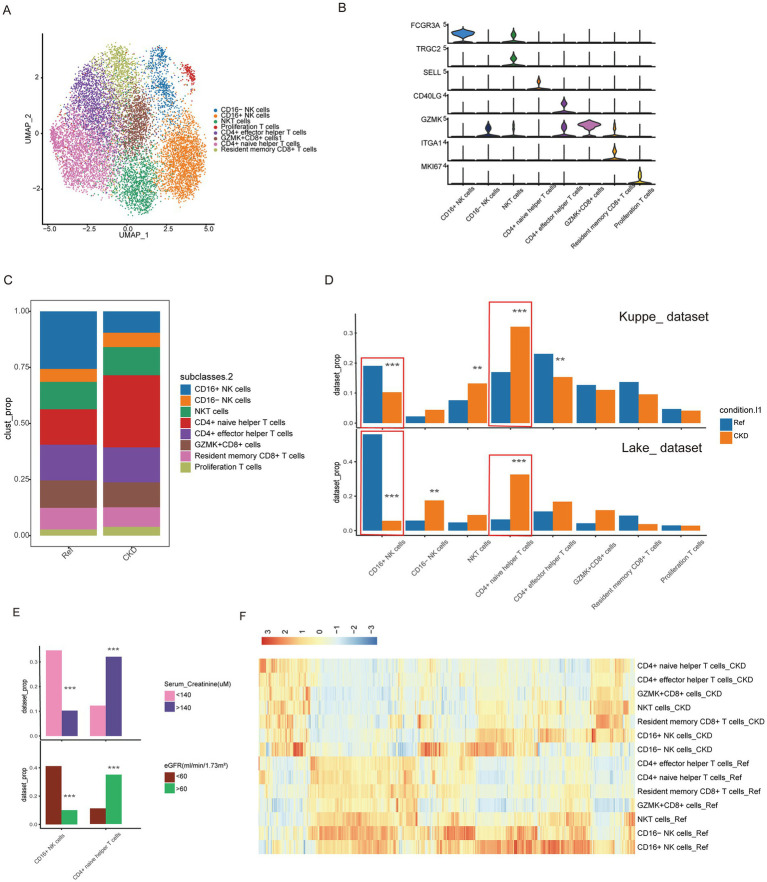
Changes in the composition and function of T cells and NK cells. **(A)** UMAP plots of different clusters. The colors of the cells represent different cell types. **(B)** Violin plot showing the expression levels of representative marker genes for 8 clusters. **(C)** Proportion of different cell populations in normal and CKD kidney tissues. **(D)** The percentages of cell clusters under different conditions were determined to exhibit significant changes based on Differential Proportional Analysis (DPA) (**Bonferroni-corrected *p* value < 0.01; ***Bonferroni-corrected p value < 0.001). **(E)** Percentage changes in cell clusters at different eGFR, and serum creatinine levels were determined according to difference proportions analysis (DPA) (***Bonferroni-corrected *p* value < 0.001). **(F)** The heatmap displays the enrichment of Hallmark and KEGG gene sets within eight T and NK cell clusters in CKD or normal kidneys.

To better understand the changes in lymphoid cells within the kidney in CKD, we performed graph-based reclustering of 11,250 subsets of T cells and NK cells. We then identified CD8 T cells, CD4 T cells and NK cells based on the expression of canonical marker genes. The CD8 T cell group comprises three subgroups, while the CD4 T cell and NK cell groups each consist of two subgroups, along with one proliferating T cell subgroup (Figure 2A; Supplementary Figures S2A,D). T cells were identified by CD3D, and TYROBP gene was strongly expressed in NK cells (Supplementary Figure S2B). CD8 T cell were identified by the expression of CD8A, LTB was used as marker gene to identify CD4 T cell (Supplementary Figure S2C). The violin plot shows the specific expression of key marker genes for each subgroup (Figure 2B).

Cell subgroups with significant changes in relative proportions may be one of the contributing factors to the occurrence and progression of chronic kidney disease. Cell relative proportions show significant changes in several cell subgroups in CKD, including CD16+ NK cells, CD4+ naive helper T cells, etc. (Figure 2C). To determine whether these changes are due to chance, we conducted permutation-based statistical test (differential proportion analysis) (19). As shown in Figure 2D, across different datasets, there are significant and consistently altered proportions, including a significant contraction of CD16+ NK cells (*p* < 0.001) and a significant expansion of CD4+ naive helper T cells (*p* < 0.001). As the primary mediators of cytotoxicity, the decrease in the CD16+ NK cell subgroup may be associated with reduced NK cell cytotoxicity and the immunosuppressive environment of CKD (23). It has been reported that the progression of human CKD is associated with an increase in the number of CD4 naive helper T cells in the kidneys (24, 25). To further understand the clinical relevance of these changes, we analyzed the association between lymphoid cell compositions and clinical parameters. When stratified by serum creatinine level, patients with impaired renal function (>140 μM) had a significantly lower proportion of CD16+ NK cells and an increased proportion of CD4+ naive helper T cells compared with patients with normal renal function (<140 μM) (*p* < 0.001) (26). Similarly, patients with impaired GFR (<60 mL/min/1.73m^2^) exhibited reduced CD16+ NK cells and increased CD4+ naive helper T cells compared to those with normal GFR (>60 mL/min/1.73m^2^) (*p* < 0.001) (Figure 2E; Supplementary Figures S2E–H). In contrast, there were no significant differences in the analyzed comparisons of age-age strata (young (<30 years), adult (30–60 years) and elderly (>60 years) groups) and gender (Supplementary Figures S2E–H). These findings suggest that alterations in CD16+ NK cells and CD4+ naive helper T cells are strongly associated with renal functional status. To investigate the functional changes of T cells and NK cells in CKD, we then used the Vision tool to perform genome enrichment analysis on relevant cell subgroups. We observed that the cellular functions of T cells and NK cells in the kidney showed relatively consistent responses to CKD (Figure 2F). Several WNT signaling pathways were activated (Supplementary Figure S2E), while genes involved in glycolysis, glucose production and fatty acid metabolism were inhibited (Supplementary Figures S2F,G). The metabolic imbalance of immune cells can lead to the accumulation of toxic metabolites, oxidative stress, and fibrosis, which are key factors in the progression of CKD (27).

While our initial analysis captured various immune cell populations, B cells were excluded from the final analysis due to technical limitations. B cells were notably underrepresented in our dataset (4,061 cells total), with only 4 out of 65 samples containing more than 30 B cells. Moreover, we observed significant sampling bias, with a single sample (CDm8) accounting for over 50% of all B cells. These technical constraints prevented reliable subclustering analysis and meaningful biological interpretation of B cell populations. Future studies specifically designed to capture adequate B cell populations will be valuable for understanding their role in CKD pathogenesis.

### 3.3 Changes in kidney myeloid cell composition and function in CKD

**Figure 3:**
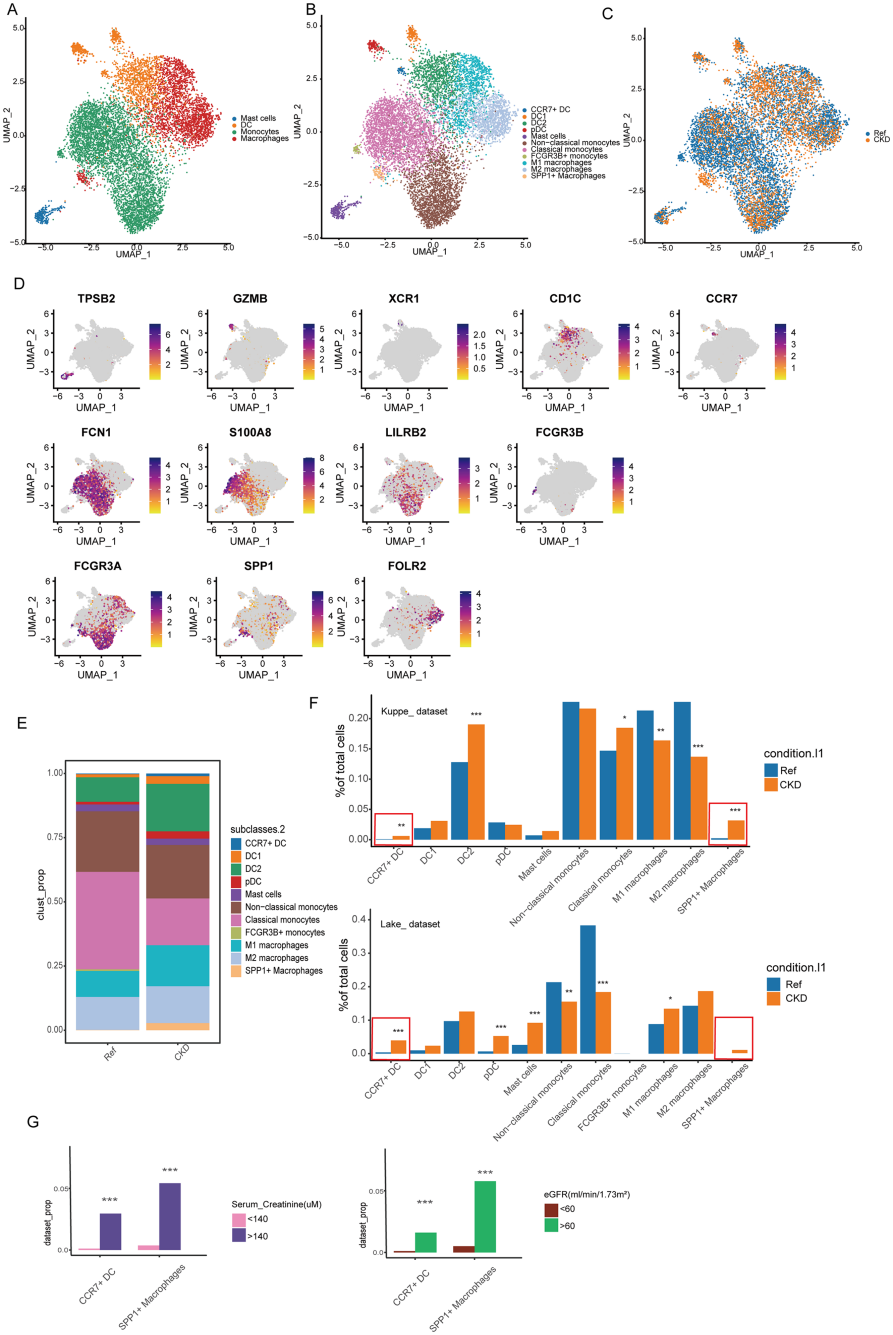
Changes in the composition of myeloid cells. **(A)** UMAP analysis was performed on myeloid cells to delineate 4 major cell populations. **(B)** UMAP plots of different clusters. The colors of the cells represent different cell types. **(C)** UMAP plots of cell clusters from different conditions. **(D)** Expression of typical marker genes in different myeloid cell clusters. **(E)** Proportion of different myeloid cell populations in normal and CKD kidney tissues. **(F)** The percentages of cell clusters under different conditions were determined to exhibit significant changes based on DPA (*Bonferroni-corrected *p* value <0.01; **Bonferroni-corrected *p* value <0.01; ***Bonferroni-corrected *p* value <0.001). **(G)** Percentage changes in cell clusters at different eGFR, and serum creatinine levels were determined according to difference proportions analysis (DPA) (***Bonferroni-corrected *p* value < 0.001).

Previous data from mice indicate that the plasticity of myeloid cells plays a crucial role in kidney injury and repair (28). To further characterize the composition and phenotypic changes of myeloid cells in CKD, we performed unbiased clustering on myeloid cells, resulting in the classification of these cells into 11 clusters. Based on the expression of typical marker genes in each cluster, they were categorized into mast cells, dendritic cells (DC), monocytes, and macrophages, with one cluster of mast cells, four clusters of DC, and three clusters each of monocytes and macrophages (Figures 3A–D). Differential proportion analysis revealed that subgroups with significant and consistent changes in composition across two independent datasets include CCR7+ DC and SPP1+ macrophages subgroups, which were scarcely detected in normal kidneys but appeared in CKD. The increased abundance of CCR7+ DC in CKD may promote migration to draining lymph nodes by binding to CCR19/CCR21 (Figures 3E,F) (29). The SPP1+ macrophages subgroup expands after organ injury, promoting the fibrotic process, and is associated with the prognosis of various diseases (30, 31). We next examined the relationship between myeloid cell populations and clinical parameters. The proportions of CCR7+ DC and SPP1+ macrophages were significantly higher in patients with renal impairment (serum creatinine > 140 μM) compared to those with normal renal function (*p* < 0.001). Similar patterns were observed when comparing patients with impaired GFR (<60 mL/min/1.73m^2^) to those with normal GFR (Figure 3G; Supplementary Figures S3A–D). In contrast, there were no significant differences in the analyzed comparisons of age-age strata and gender (Supplementary Figures S3A–D). The association between these clinical parameters and bone marrow cell composition provides additional evidence for a link between immune cell alterations and CKD.

**Figure 4:**
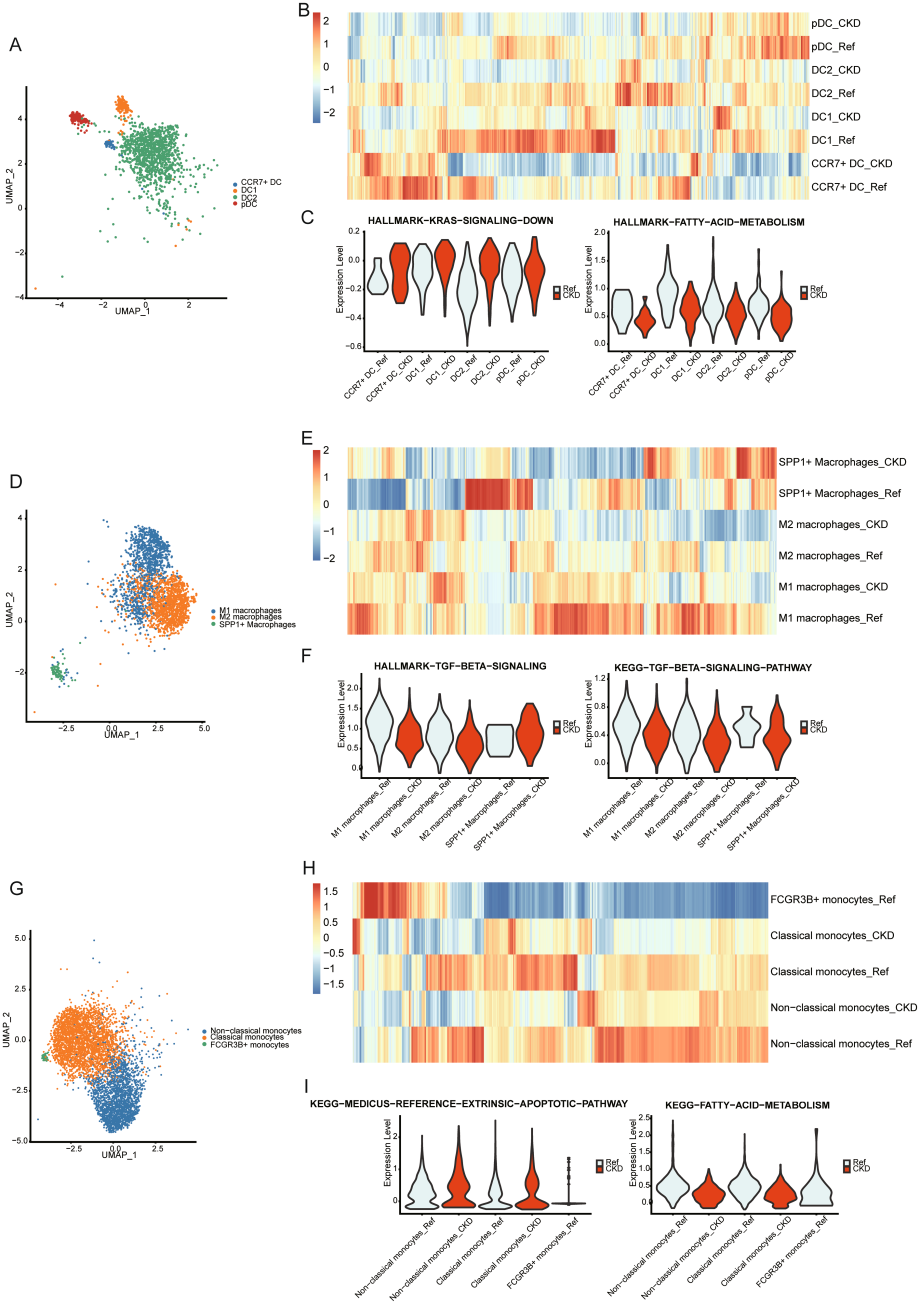
Changes in the function of myeloid cells. **(A)** Umap plot of DC subpopulations. **(B)** The heatmap displays the enrichment of Hallmark and KEGG gene sets within 4 DC subpopulations in CKD or normal kidneys. **(C)** Violin plots showing enrichment of KRAS down-regulated genes (left panel), fatty acid metabolism genes (right panel) in the DC subpopulation of normal and CKD kidneys. **(D)** Umap plot of macrophages subpopulations. **(E)** The heatmap displays the enrichment of Hallmark and KEGG gene sets within 3 macrophages subpopulations in CKD or normal kidneys. **(F)** Violin plots showing enrichment of TGF-BETA signaling pathway in the macrophage subpopulation of normal and CKD kidneys. **(G)** Umap plot of monocytes subpopulations. **(H)** The heatmap displays the enrichment of Hallmark and KEGG gene sets within 4 monocytes subpopulations in CKD or normal kidneys. **(I)** Violin plots showing enrichment of extrinsic apoptosis pathway genes (left panel), fatty acid metabolism genes (right panel) in the monocyte subpopulation of normal and CKD kidneys.

We subsequently compared changes in pathway enrichment levels among DC, monocyte, and macrophage subgroups in CKD. In CKD, the enrichment level of genes downregulated due to KRAS activation increases in the four DC subgroups, whereas genes associated with fatty acid metabolism exhibit decreased enrichment levels (Figures 4A–C). Additionally, genes related to the TGF-BETA signaling pathway are downregulated in the three macrophage subgroups (Figures 4D–F). Furthermore, genes associated with the extrinsic apoptosis pathway are upregulated in the three monocyte subgroups, while genes related to fatty acid metabolism exhibit downregulation (Figures 4G–I). The observed changes in signaling pathways suggest their potential involvement in CKD pathophysiology.

### 3.4 Interaction network between SPP1+ macrophages and injured cells in CKD

Complex tissues like the kidney rely on cell–cell communication networks to coordinate physiological functions. SPP1 macrophages increase in CKD, whereas they are rarely detected in normal kidneys. To investigate the role of SPP1 macrophages in CKD, we utilized CellChat to explore potential communication pathways between SPP1 macrophages and damaged cells.

**Figure 5:**
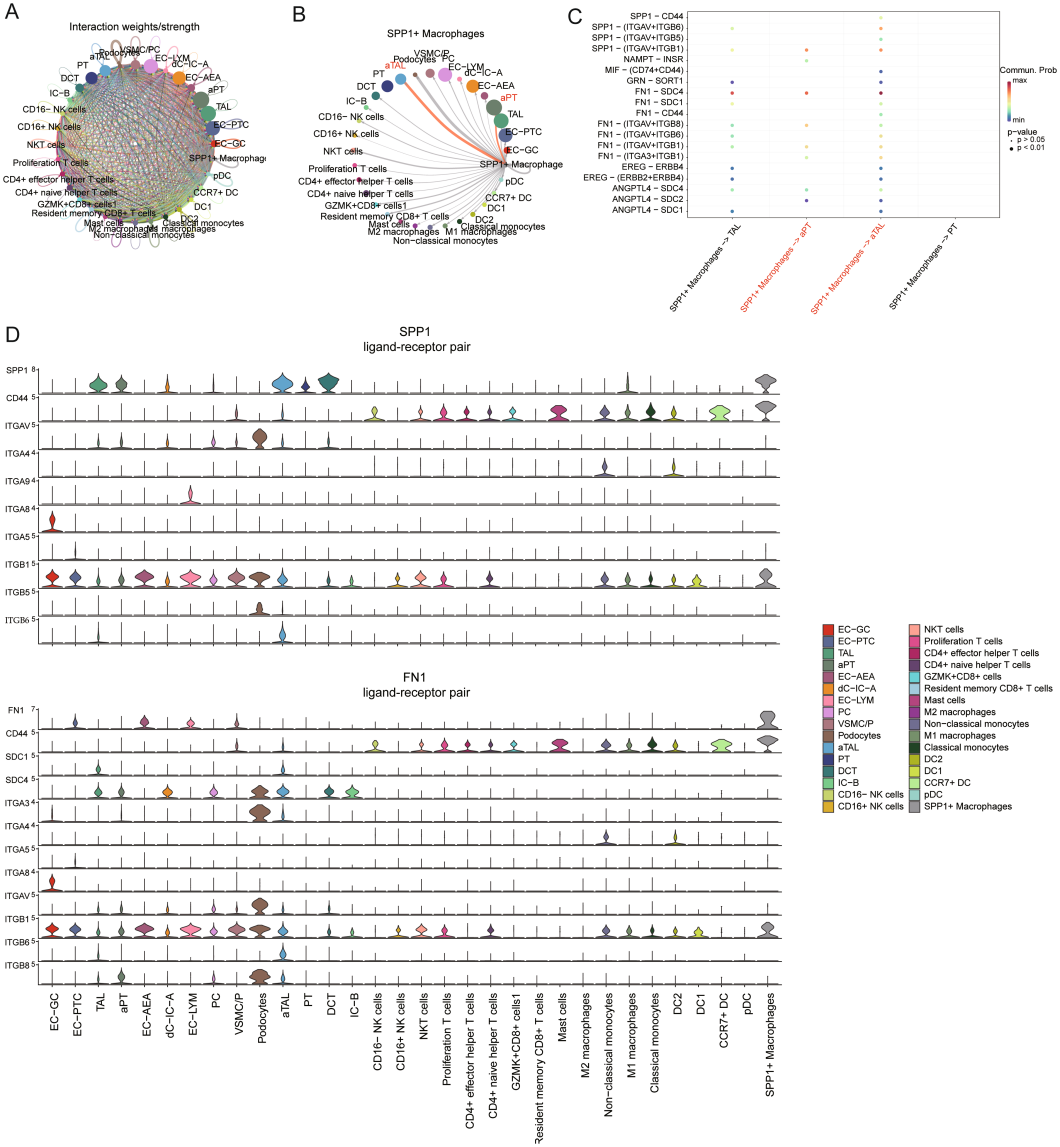
Regulation of damaged cells by SPP1 macrophages in CKD. **(A)** Circle plot showing the strength of interactions between cell clusters in CKD. **(B)** Circle plot displays the interaction strength between SPP1 macrophages as signal senders and other types of cells in CKD. The thickness of the edges line represents the strength of the interactions. **(C)** The bubble plot displays all significant ligand-receptor interactions from SPP1 macrophages to PT, ATL and corresponding damaged cells. **(D)** Violin plots showing the expression of signaling genes SPP1 and FN1 in each cellular subgroup.

We first assessed the strength of cell–cell communication networks aggregated in CKD (Figure 5A), revealing a widespread network of communication among cells. Next, we examined the signals emitted by SPP1 macrophages (Figure 5B), and the results showed that aPT, aTAL, and podocytes received the strongest signals. This suggests that SPP1+ macrophages primarily target damaged cells in CKD for regulation. We further investigated the cellular communication of specific ligand-receptor pairs between SPP1+ macrophages and damaged cells (aPT and aTAL) (Figure 5C). The results showed 19 ligand-receptor pairs including SPP1, NAMPT, MIF, GRN, FN1, EREG and ANGPTL4 signaling pathways were involved in the communication from SPP1+ macrophages to damaged cells, with the ligand SPP1, FN1 and its multi-subunit receptors acting as the major signals (Figure 5C) and genes associated with SPP1 and FN1 signaling exhibit high expression in damaged cells (Figure 5D). The receptor-ligand related genes of the SPP1 and FN1 signaling pathways are associated with the expression of the extracellular matrix (32). Excessive accumulation of the extracellular matrix is closely related to fibrosis (33), which may be one of the pathways through which SPP1+ macrophages promote organ fibrosis.

**Figure 6:**
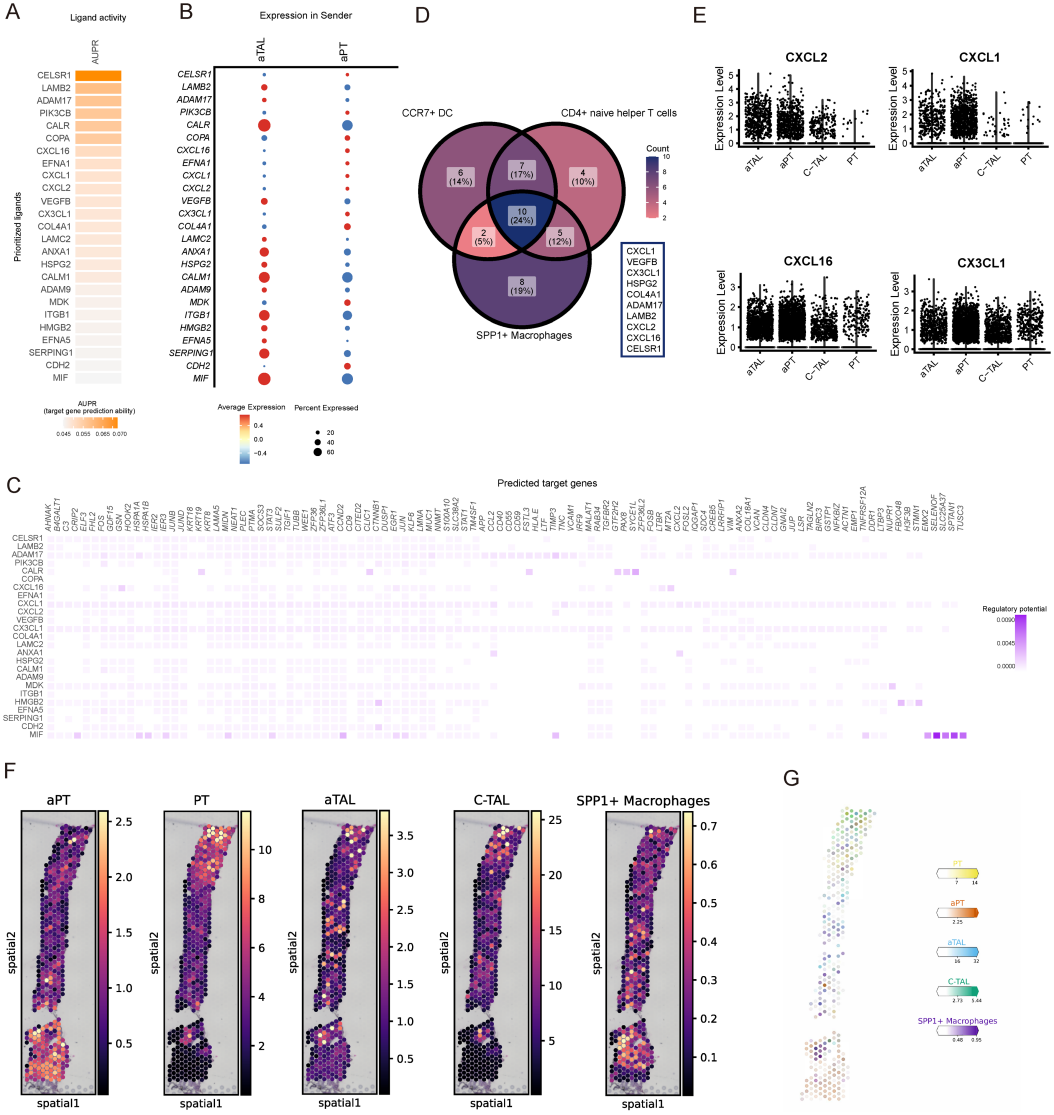
Subpopulations of kidney aPT and aTAL cells recruit spp1+ macrophages by releasing chemokines. **(A)** NicheNet-predicted ligand activity when using spp1 macrophages as receiver cells. **(B)** NicheNet-predicted expression of ligand genes in aTAL and aPT. **(C)** NicheNet’s ligand-target matrix, represents the potential for regulation between ligands and target genes during communication between kidney-damaged cells and SPP1 macrophages. **(D)** Venn diagram of the list of ligand activities predicted by NicheNet when using different increased immune cells as receiver cells. **(E)** Violin plots showing expression of different chemokines in injured and normal cell types. **(F)** Spatial image of cell abundance estimated by cell2location for selected cell types (color intensity). **(G)** Estimated cell abundance (color intensity) of the cell type (color) shown on the image of the same CKD kidney sample. Scale bar is 1 mm.

A substantial body of literature indicates that damaged renal cells recruit immune cells (34–36). Therefore, we applied NicheNet to explore the potential mechanisms by which damaged cells in CKD recruit immune cells (37). NicheNet integrates gene expression data with pre-existing signaling and gene regulatory network models to predict ligand interactions within the sending cells. These interactions are not limited to homologous receptors but extend to any potential downstream genes. In our analysis, we identified aPT and aTAL as the sending centers and defined target gene sets using the differentially expressed genes of aPT and aTAL compared to normal PT and TAL. NicheNet analysis was performed with the increased immune cell types as the receivers. The analysis of SPP1+ macrophages showed a higher ligand activity measure for CELSR1, LAMB2, and ADAM17 (Figure 6A), and the expression levels of these ligands were comparable in aPT and aTAL (Figure 6B). We subsequently inferred the target genes of these ligand-receptor interactions (Figure 6C). Lastly, we examined the intersection of ligand lists for the increased immune cells and identified 10 overlapping ligands (Figure 6D), including several chemokines responsible for immune cell infiltration. Notably, the expression of chemokines CXCL2, CXCL1, CXCL16, and CX3CL1 was increased in both aPT and aTAL (Figure 6E). Recent studies have indicated that damaged PT cells release CXCL1, which attracts immune cells and promotes the progression of renal fibrosis (38). Therefore, the upregulation of chemokines in damaged cells in CKD may represent a key mechanism for the recruitment of various immune cells. To further validate the interaction between SPP1+ macrophages and kidney injury cells, we identified the spatial distribution of SPP1 macrophages and kidney injury cells in combination with spatial transcriptome data. The results showed regional co-localization of SPP1 macrophages with kidney injury cells (Figures 6F,G), suggesting that there is recruitment of these kidney injury cells to SPP1 macrophages and possible interaction between the two cell populations.

## 4 Discussion

Increasing evidence suggests that immune dysfunction is one of the key factors contributing to the occurrence and progression of CKD, known as secondary immunodeficiency related to kidney disease (SIDKD) (39). While progress has been made in understanding the immune mechanisms of kidney disease using mouse models (5, 40), their translation to human conditions requires further validation. This is because mice, particularly those bred under specific pathogen-free conditions, may have immune and inflammatory environments different from humans. Currently, the composition and changes of immune cells in the kidneys of human CKD patients remain undetermined compared to mouse kidneys.

In this study, we integrated scRNA data from human CKD patients and normal kidney tissues to systematically analyze the composition and functional changes of immune cells, aiming to construct a comprehensive profile of the kidney immune system in CKD patients. By integrating and analyzing two independent datasets, we could effectively mitigate experimental biases arising from variations in experimental protocols, technical differences, limited sample sizes, and individual variations. In terms of changes in cellular composition, compared to immune cells in normal kidneys, CD16+ NK cells decrease, while CD4+ naive helper T cells and CCR7+ DC increase in the kidneys of CKD patients. In addition, SPP1+ macrophages are a unique cell type in CKD. In terms of cellular functional changes, the responses of T cells and NK cells in the kidney to CKD are relatively consistent, including activation of the WNT pathway and inhibition of some metabolic pathways. Functional changes in myeloid cell subsets vary in CKD, but there are also abnormalities in metabolic pathways. Some emerging therapies aim to achieve therapeutic goals by modulating the metabolic status of immune cells in the disease (41). Targeting immune metabolism is a promising approach to the treatment of kidney disease. It is noteworthy that we observed cell-to-cell communication between SPP1 macrophages and injured cells in CKD, promoting cellular fibrosis through signal transmission via SPP1, FN1, and multiple receptors. Our findings align with existing literature demonstrating the pro-fibrotic role of SPP1+ macrophages in other organ systems, particularly in myocardial infarction (30). This cross-organ consistency lends additional credibility to our observations. The interaction between injured cells and SPP1 macrophages should be considered as a potential therapeutic target for CKD treatment.

Our findings have several potential therapeutic implications. Firstly, the pathways involving relevant chemokines associated with CKD damage could be targeted for intervention. Currently, clinically approved drugs targeting chemokines include anti-CCR4 antibodies (Mogamulizumab) and CXCR4 antagonists (Plerixafor, AMD3100), which are utilized in the treatment of hematological malignancies. Additionally, there are ongoing efforts to develop various therapeutic strategies targeting different chemokine receptor-ligand axes, which have shown considerable promise and are currently in clinical development. Secondly, the depletion of SPP1+ macrophages through antibody-based or cellular therapies should also be considered.

To further validate the mechanisms identified, future studies should experimentally test the chemokine pathways observed in our analysis, such as the CXCL1-CXCR2 and CXCL16-CXCR6 axes, which are key in immune cell recruitment. Targeting these pathways using antagonists or knockout models could confirm their role in CKD progression. Additionally, SPP1+ macrophages, identified as a critical pro-fibrotic cell type, warrant further investigation through depletion strategies or receptor-ligand interaction studies. These experiments could provide valuable insights into immune-mediated fibrosis and novel therapeutic targets.

In summary, we have established a map of human CKD renal immune cells based on integrated analysis of different scRNA-seq data. This map delineates the detailed profiles of immune cells in the CKD renal microenvironment and reveals the potential value of therapeutic strategies targeting CKD-injured cells, SPP1 macrophages, or molecules involved in their crosstalk. These findings greatly contribute to our understanding of the heterogeneity of immune cells in CKD kidneys and the complexity of the renal microenvironment. Nevertheless, the cellular communication between CKD-damaged cells and SPP1 macrophages, as well as the mechanism by which SPP1 macrophages promote renal fibrosis, remain to be elucidated in future studies. Our current findings provide a solid foundation for future mechanistic studies. Furthermore, it is important to acknowledge that our current atlas lacks detailed B cell profiling due to technical limitations in cell capture and representation. This constraint affects our comprehensive understanding of adaptive immunity in CKD, particularly regarding antibody-mediated responses and B cell interactions with other immune populations. Future studies employing B cell-specific enrichment protocols would be valuable to complement our current findings and provide a more complete picture of immune responses in CKD. Additionally, disease activity, disease stage, and disease duration may have a significant impact on the composition and behavior of immune cells in CKD, which are not included in the current data. Therefore, further exploration of cellular composition and behavior at different stages of CKD is warranted in future research. Specifically, future studies should prioritize comprehensive clinical data collection to establish more robust connections between immune cell profiles and disease progression. Longitudinal sampling tracking immune cell changes would be particularly valuable, as it could reveal dynamic shifts in immune populations that correlate with disease advancement. Integration of standardized disease activity markers, precise staging criteria, and detailed treatment histories would help better understand therapeutic impacts on immune cell compositions. Moreover, comprehensive metabolic profiles, inflammatory markers, and comorbidity data could provide crucial context for interpreting immune cell alterations. Such multi-parameter analyses could potentially identify stage-specific immune signatures and reveal novel therapeutic targets. Understanding these correlations between immune cell dynamics and clinical outcomes could ultimately facilitate the development of more targeted therapeutic strategies for CKD patients.

## Data Availability

The original contributions presented in the study are publicly available. The datasets used in this study can be found in the following repositories: Kuppe et al.’s data at Zenodo (https://doi.org/10.5281/zenodo.4059315), Lake et al.’s data at GEO (accession number GSE183279), and Stewart et al.’s data at www.kidneycellatlas.org.
