# Supplementary material for: Single-cell transcriptome analysis reveals status changes of immune cells in chronic kidney disease
Source: Front Med (Lausanne). 2024 Dec 3;11:1434535. doi: 10.3389/fmed.2024.1434535 (PMC11649435; doi:10.3389/fmed.2024.1434535)
Supplement: Supplementary file 2 [file Data_Sheet_2.docx]

**Supplementary Information**

Single-cell transcriptome analysis reveals status changes of immune cells in chronic kidney disease

Xinhuan Fan^†,1^, Yuxin Zhu^†,2^, Hao Kan^2,3^, Aiqin Mao^2,3^, Li Geng^2^, Changzhu Li^2,3^, Ka Zhang ^*,2,3^

^1^Department of Urology, Lu'an Hospital of Anhui Medical University, Lu'an, 237005, China.

^2^Wuxi School of Medicine, Jiangnan University, Wuxi, 214000, China

^3^School of Food Science and Technology, Jiangnan University, Wuxi, 214000, China.

^†^These authors contributed equally to this study.

*** Correspondence:**

Ka Zhang: zh0818k@163.com; Wuxi School of Medicine, School of Food Science and Technology, Jiangnan University, Wuxi, China

**Figure S1**


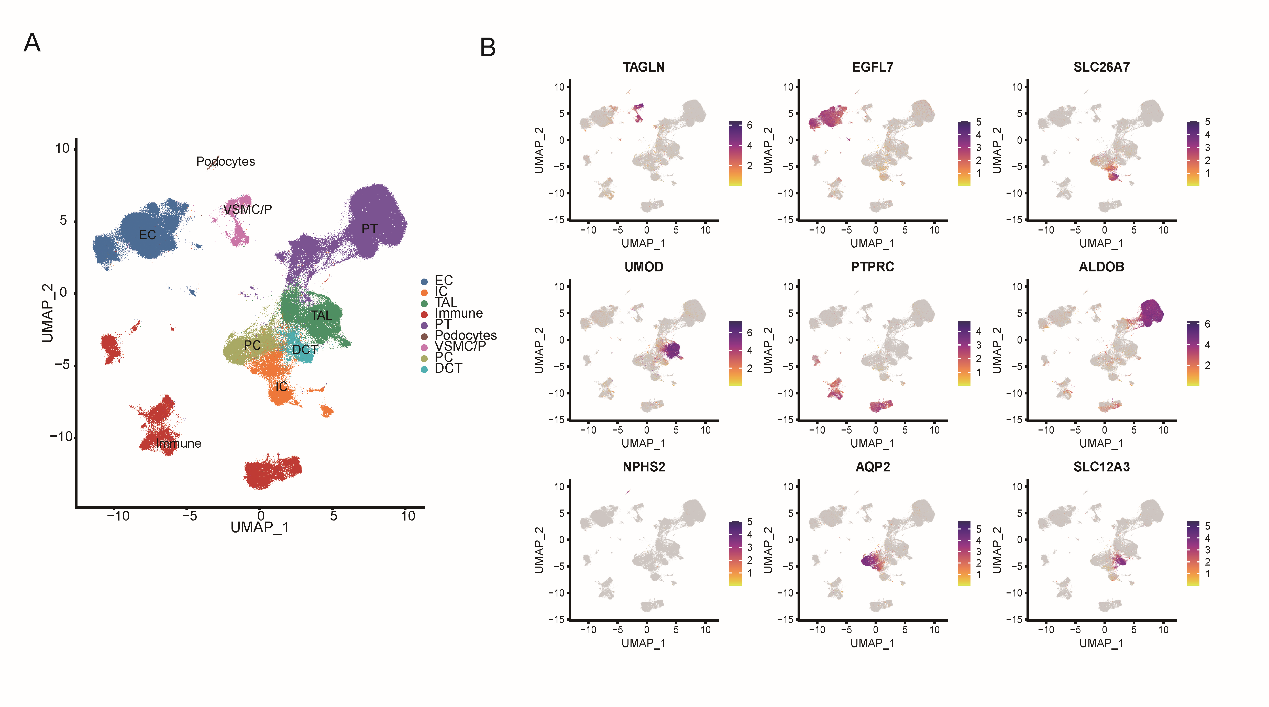


**Figure S1:** **Expression levels of representative markers. A,** UMAP analysis of normal and CKD kidney cell atlases delineating 9 major cell clusters. EC, endothelial cell; IC, intercalated cell; TAL, thick ascending limb; PT, proximal tubule; VSMC/P, vascular smooth muscle cell; PC, principal cell; DCT,distal convoluted tubule. **B**, Normalized expression of marker genes in major cell types.

**Figure S2**


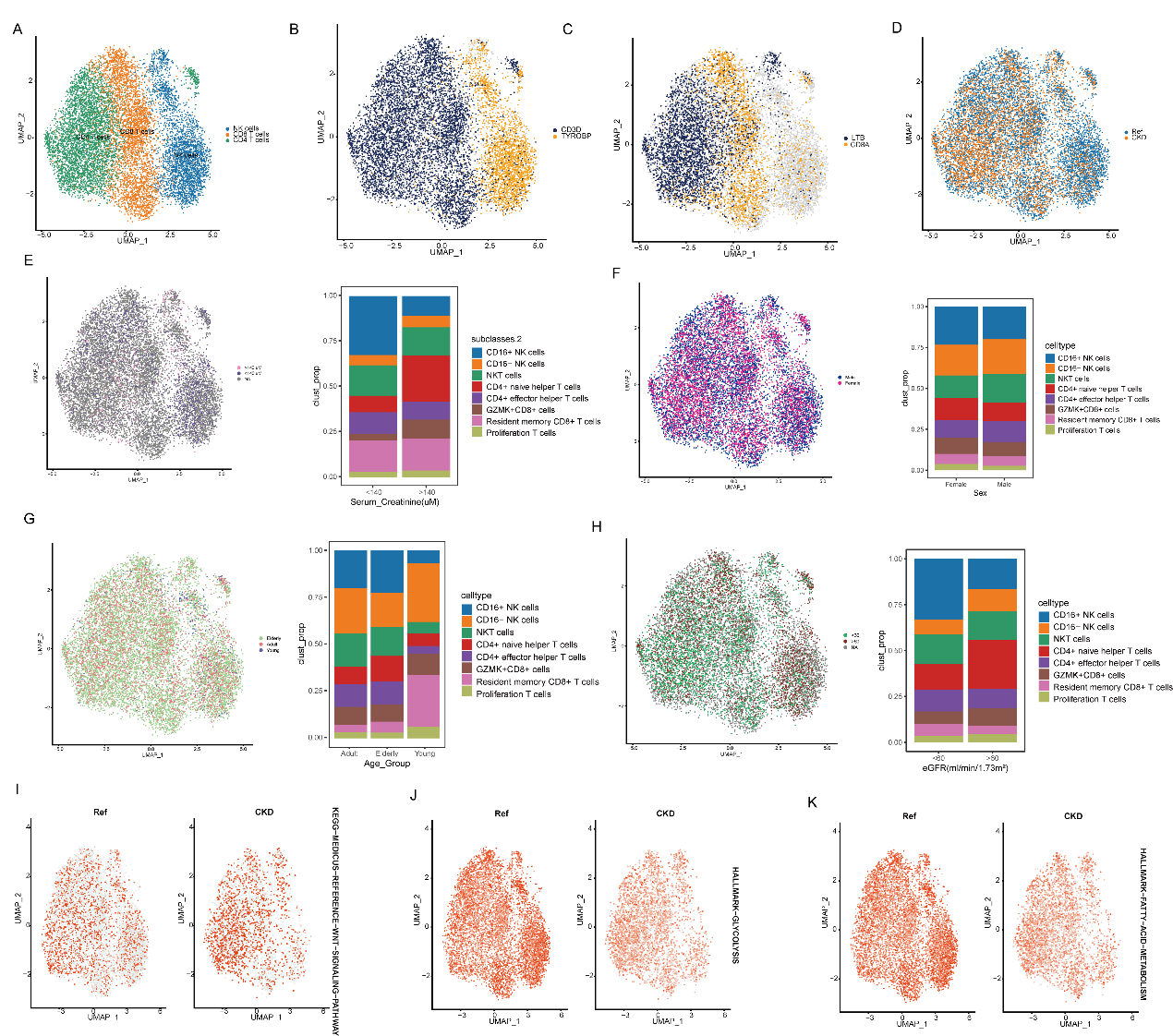


**Figure S2.** **Subgroup analysis of T-cell clinical data.** **A**, UMAP analysis was performed on T cells and NK cells to delineate 3 major cell populations. **B**, Normalized expression of T cell and NK cell marker genes. T cell (CD3D), NK cell(TYROBP). **C**. Normalized expression of CD4+ T cell and CD8+ T cell marker genes. CD4+ T cell (LTB), CD8+ T cell (CD8A). **D-H**, UMAP plots and proportions of cell populations under different conditions, serum creatinine (>140 uM: renal impairment; <140 uM: healthy), gender, age (<30 years: young; 30<&>60 years: adults; >60 years: elderly) and GFR (>60 uM: renal impairment; <60 uM: healthy). **I-K,** In normal or CKD kidneys, UMAP demonstrates gene enrichment of the WNT signaling pathway (I), glycolysis (J), and fatty acid metabolism (K).

**Figure S3**


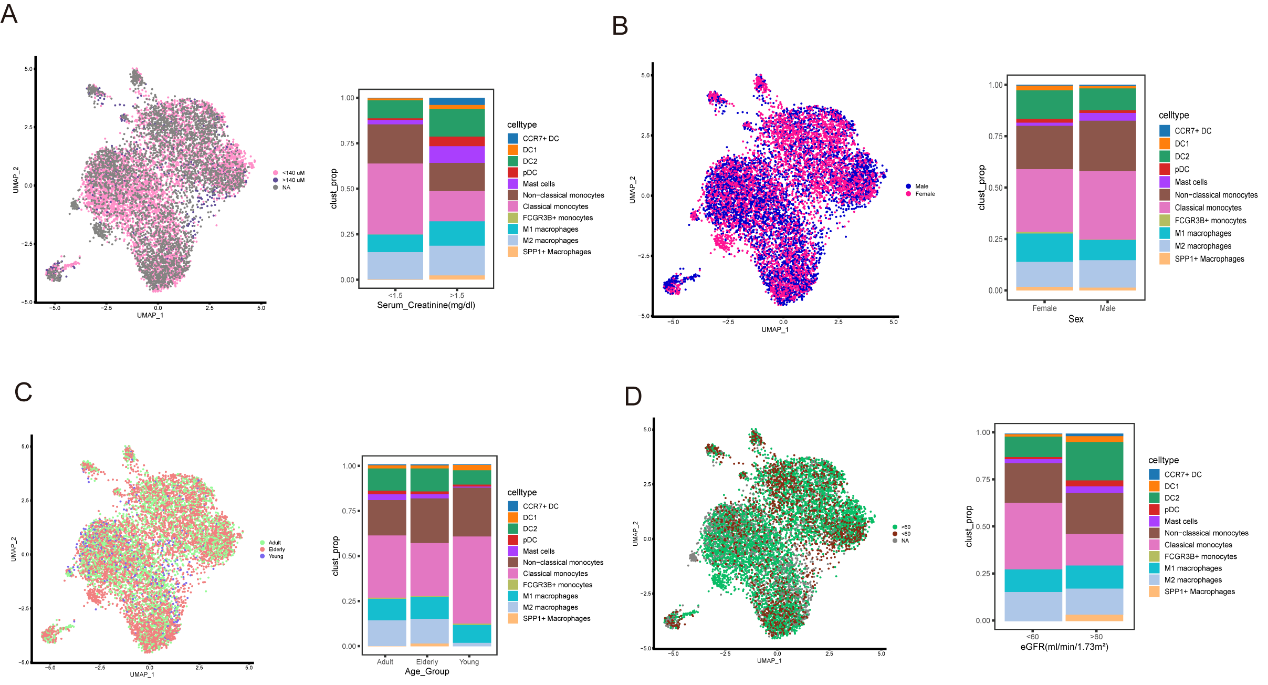


**Figure S3** **Subgroup analysis of myeloid cell clinical data. A-D**, UMAP plots and proportions of cell populations under different conditions, serum creatinine (>140 uM: renal impairment; <140 uM: healthy), gender, age (<30 years: young; 30<&>60 years: adults; >60 years: elderly) and GFR (>60 uM: renal impairment; <60 uM: healthy).
